# Temporal trend of congenital syphilis in the most populous municipality of metropolitan region II of Rio de Janeiro state

**DOI:** 10.1590/1984-0462/2023/41/2021337

**Published:** 2023-03-13

**Authors:** Lidiane Santos de Morais, Sandra Vitoria Thuler Pimentel, Helia Kawa, Sandra Costa Fonseca

**Affiliations:** a Universidade Federal Fluminense, Niterói, RJ, Brazil.

**Keywords:** Syphilis, congenital, Health information systems, Time series studies, Healthcare disparities, Sífilis congênita, Sistemas de informação em saúde, Estudos de séries temporais, Disparidades em assistência à saúde

## Abstract

**Objective:**

This study aimed to explore the temporal trend in congenital syphilis, according to sociodemographic and prenatal care in the city of São Gonçalo – Rio de Janeiro, from 2007 to 2018.

**Methods:**

Ecological time series study, with data from SINAN (Information System for Notifiable Diseases) and SINASC (Information System on Live Births databases). We calculated annual incidence (per 1,000 live births) according to sociodemographic and prenatal variables. For the same variables, we calculated trends by logarithmic regression (*Joinpoint Regression*), estimating the annual percentage change.

**Results:**

A total of 2,420 cases were reported from 2007 to 2018, with an increasing trend: 64.9% per year (2010–2013) and 24.9% (2013–2018). In 2018, the highest rates were in adolescents (90.6/1,000 live births), black women (87.6/1,000 live births), low-educated women (122.8/1,000 live births), and those without prenatal care (677.4/1,000 live births). The annual percentage change of these categories was, respectively, 37.3% (2010–2018), 33.5% (2012–2018), 39.9% (2014–2018), and 85.0% (2011–2015), but all categories showed a crescent trend.

**Conclusions:**

We identified high congenital syphilis incidences and crescent trends, especially in more vulnerable groups, pointing to social and healthcare inequalities. Prenatal care needs to be more comprehensive and qualified, primarily for young, low-educated, and black women.

## RESUMO

**Objetivo:**

Explorar a tendência da sífilis congênita segundo características sociodemográficas e pré-natal, no município de São Gonçalo, Rio de Janeiro, no período de 2007 a 2018.

**Métodos:**

Estudo ecológico de série temporal da incidência de sífilis congênita, com dados do Sistema de Informação de Agravos de Notificação (SINAN) e do Sistema de Informações sobre Nascidos Vivos (SINASC). A incidência anual, por mil nascidos vivos, foi calculada segundo variáveis sociodemográficas e pré-natal. Para as mesmas variáveis, foi estimada a série temporal por regressão logarítmica (Joinpoint Regression). A medida de tendência foi a variação percentual anual.

**Resultados:**

Notificaram-se 2.420 casos de 2007 a 2018, com tendência crescente: 64,9 ao ano (2010–2013) e 24,9% (2013–2018). Em 2018, a taxa de sífilis congênita atingiu 41,6/1.000 nascidos vivos, sendo as maiores taxas em adolescentes (90,6/1.000 nascidos vivos), mulheres de cor preta (87,6/1.000 nascidos vivos), com baixa escolaridade (122,8/1.000 nascidos vivos) e aquelas sem pré-natal (677,4/1.000 nascidos vivos). As variações percentuais anuais dessas categorias foram, respectivamente, 37,3 (2010–2018), 33,5 (2012–2018), 39,9 (2014–2018) e 85% (2011–2015), e as demais categorias também tiveram incremento.

**Conclusões:**

Identificaram-se incidência elevada e tendência crescente de sífilis congênita em São Gonçalo, principalmente em grupos mais vulnerabilizados, apontando desigualdades em saúde. O pré-natal precisa ser ampliado quantitativa e qualitativamente, sobretudo para mulheres jovens, negras e de baixa escolaridade.

## INTRODUCTION

Congenital syphilis (CS) is still an important public health problem in the world and in Brazil.^1,2^ In the Americas, some countries have already achieved their elimination.^3^ In 2015, Cuba became the first country in the world to receive validation from the World Health Organization (WHO) for the elimination of vertical transmission of the acquired immunodeficiency virus (HIV) and syphilis.^3^

However, in Brazil, the target of 0.5 or fewer cases of CS per 1,000 live births (LB) is far from being achieved, considering the incidence of 8.2 new cases per 1,000 LB recorded in 2019 and the increasing trend in recent years.^2,4^

Among the Brazilian states, Rio de Janeiro held the highest incidence of CS in 2019, reaching 20.1 per 1,000 LB, and the highest detection of syphilis in pregnant women, 44.5 per 1,000 LB.^2^ According to the 2018 state bulletin on syphilis, the most compromised health regions are the metropolitan regions I and II.^5^ There are more studies on CS in metropolitan region I, concentrated in the state capital.^6–10^ An ecological study from 2011 to 2014 in the city of Rio de Janeiro identified an incidence of 17.3 per 1,000 LB. The variable that showed greater association with CS was the low percentage of pregnant women with seven or more prenatal consultations,^9^ corroborating previous results.^7,8^ From 2013 to 2017, the reported incidence was 19.6 per 1,000 LB, and the shortage of benzathine penicillin was an additional factor related to the increase in cases.^10^

In metropolitan region II, we identified only one population-based study, in Niterói, which showed increasing incidences, reaching 23.2 per 1,000 LB in 2016, more accentuated in magnitude and trend in adolescents, women of low education, brown, and black.^11^ There are no studies on the municipality of São Gonçalo, which is the most populous in the region and the second in the state of Rio de Janeiro, with about one million inhabitants. Local studies are important because there is variation in the pattern of occurrence of diseases and illnesses of the population, in the settings of the health system, use of services, and its own policies with different models of organization of health care. The results at the municipal level can contribute by identifying differentiated problems and defining inequalities, so relevant in the study of syphilis, to support planning and resource allocation.^12^

The objective of this study was to analyze the magnitude, temporal distribution according to sociodemographic status, and infant mortality related to CS in the most populous municipality of the metropolitan region II of the state of Rio de Janeiro, São Gonçalo, from 2007 to 2018. Additionally, the quality of the completion of CS investigation forms was described. Our hypothesis is that São Gonçalo contributes to the high incidence of CS in Rio de Janeiro and unequally affects its female residents, according to sociodemographic variables.

## METHOD

Time series study, with population data from São Gonçalo, located in the metropolitan health region II of the state of Rio de Janeiro. Located 22 km from the capital city of Rio de Janeiro, it has 1,077,687 inhabitants, a municipal human development index (MDI) of 0.739, infant mortality of 14.5 per 1,000 LB, and 194 health facilities.^13^

The population base was composed of all LB from 2007 to 2018, and CS cases included LB, abortions, and stillbirths. Data from the Notifiable Diseases Information System (SINAN), made available in unidentified files, by the State Health Secretariat of Rio de Janeiro (SES-RJ) were used. To calculate the incidence of CS in each year, the number of new cases was divided by the number of LB in the same year, multiplied by 1,000. The number of LB was obtained from the Live Births Information System (SINASC), available on the SES-RJ website.^14^

The variables analyzed were as follows: skin color (mother/baby), maternal characteristics (e.g., age and education), and the number of prenatal visits. Sociodemographic and prenatal variables were categorized according to the SINASC model and the availability of SINAN data. For skin color, the categories white, black, yellow, brown, and indigenous were included. Maternal age was classified into three categories: 10–19 years, 20–34 years, and 35 years or older; schooling was classified into four categories: 0–3 years, 4–7 years, 8–11 years, and 12 years or older; and prenatal care, according to the number of consultations: 0, 1–3, 4–6, and 7 or more.

First, descriptive statistics of the percentage distribution by age, education, skin color, and the number of prenatal consultations of women with LB residing in the municipality were performed, variables used in the analysis of the incidence of CS.

Next, the annual incidence of CS was calculated, total, and according to the variables age, education, skin color, and prenatal consultations, from 2007 to 2018.

The percentage distribution of cases of CS was described, according to the final diagnosis – recent or late CS, abortion or stillbirth, and death. Regarding CS mortality in children under 1 year and fetal mortality, in addition to data from the investigation forms, we obtained data from the Mortality Information System (SIM). We calculated CS mortality by dividing the number of deaths by CS in children under 1 year by the number of LB and multiplying by 100,000. We calculated triennial values, due to the low absolute number of deaths.

The score proposed by Romero and Cunha was used to analyze the completeness of the variables of the research form, from which the variables regarding CS were taken.^15^ The cutoff points of this score to classify the quality, according to the percentage of incompleteness, are as follows: excellent (<5%), good (5–10%), regular (10–20%), poor (20–50%), and very poor (≥50%).

For the time series analysis, the annual percentage variation (APV) of the annual incidences was calculated globally and according to the sociodemographic and prenatal variables by the Monte Carlo permutation method, using the Joinpoint Regression (Statistical Research and Applications Branch, National Cancer Institute, USA), of public use. The modeling employed by Joinpoint uses log-transformed values to identify inflection points (joinpoints). When there is an inflection point where the direction reverses or different trend patterns are observed, the periods are analyzed separately. In this situation, the final year of a period always coincides with the initial year of the next one, and for each variable or category, these periods may be different in relation to the years analyzed, depending on the moment when the change is detected. If there is no change, the period is analyzed in its entirety.

For the incidence of CS and the time series, some variables were recategorized. The maternal education variable was regrouped into two categories: low (<8 years of study) and high (≥8 years of study). This choice was due to the small number of women in the zero to 3 years of study category and the similar behavior of the categories 8–11 and 12 or more years of study regarding the incidence of CS. For the variable skin color, the three categories that represent 99.8% of the population were analyzed: white, black, and brown. Finally, prenatal care was evaluated in the categories yes and no, since SINAN provides only this information on prenatal care, with yes being equivalent to any number of consultations.

This study is part of the project “Indicators of women’s and children’s health in the health regions of the state of Rio de Janeiro.” One of the objectives of the project is to evaluate CS in the different municipalities of the state. The project was approved by the Research Ethics Committee (CEP) of the Faculdade de Medicina da Universidade Federal Fluminense, opinion no. 4.091.556, on June 16, 2020. As it is a secondary database without nominal identification, the CEP dispensed the application of the informed consent form. The research complied with the ethical and scientific requirements of Resolution No. 466/12.

## RESULTS

From 2007 to 2018, 139,143 newborns (NBs) were registered in São Gonçalo. Most mothers were aged between 20 and 34 years, but there was an increase in the proportion of those aged 35 years or more and a decrease in the percentage of adolescents (Table 1). The majority of the women had studied from 8 to 11 years, with a drop in the low schooling level (0 to 7 years) throughout the period. In the first two trienniums of the period the number of white and mixed-race women was almost equal, but the percentage of mixed-race women increased, reaching almost double that of white women in the last triennium. The percentage of black women has always been much lower. Most women had seven or more prenatal visits, but in the last two trienniums, the percentage was less than 70% (Table 1).

**Table 1.** Percentage distribution of sociodemographic and prenatal characteristics of mothers of live births in São Gonçalo, RJ, from 2007 to 2018.

	2007–2009	2010–2012	2013–2015	2016–2018
Live births	34,449	34,791	36,251	33,649
Age range (years)				
10–19	18.7	18.5	18.1	16.5
20–34	71.4	70.7	69.7	69.5
≥35	9.9	10.7	12.2	14.1
Education (years of study)				
0–3	5.3	3.8	2.9	1.9
4–7	24.9	21.3	17.7	16.2
8–11	47.3	56.5	65.4	64.6
≥12	24.9	19.7	15.1	18.1
Skin color				
Caucasian	48.9	47.4	38.4	33.2
Black	2.7	4.4	6.6	4.5
Yellow skin	<0.1	0.1	0.1	0.1
Brown skin	47.7	47.1	53.8	61.3
Indigenous	<0.1	<0.1	<0.1	<0.1
Prenatal (consultations)				
Did not	1.4	1.7	1.1	0.7
1–3	4.7	4.6	5.1	5.9
4–6	21.2	19.8	25.2	26.0
≥7	72.1	72.7	66.6	66.9

Source: Information System on Live Births (SINASC).^14^

Considering the date of diagnosis, from 2007 to 2018, there were 2,420 cases of CS in the municipality of São Gonçalo. Regarding the completeness of data in the CS investigation forms, the maternal variables education and occupation were classified as bad, with the former having almost 30% of ignored or empty information. The variables treatment of the pregnant woman and her partner were rated as regular. The others were between good and excellent. Regarding the quality of the NB data, there was one very bad variable (quantitative of CSF Venereal Disease Research Laboratory [VDRL]=99% of ignored or empty information), two bad ones (quantitative of serum VDRL=22.3% and long-bones radiographs =23.2%), and the others were excellent (Table 2). We highlight the great improvement in the completion of the maternal education variable over the period: from 2014 to 2018, incompleteness was 14% (data not shown in the table).

**Table 2.** Quality of filling in the fields of the congenital syphilis investigation form, in São Gonçalo, RJ, from 2008 to 2017.

	Field classification	Ignored or empty (%)	Quality
Maternal variables			
Age	Mandatory	1	Great
Education	Essential	28.1	Not good
Color/race	Essential	5.7	Good
Occupation	No mandatory	22.6	Not good
Prenatal	Mandatory	6.3	Good
Maternal diagnosis	Mandatory	2.4	Great
Maternal treatment	Mandatory	17.5	Regular
Partner treatment	Essential	17.5	Regular
Maternal VDRL on admission	Mandatory	4.2	Great
Final diagnosis	Mandatory	0	Great
Evolution	Mandatory	1.1	Great
NB variables			
NB’s VDRL	Mandatory	1.8	Great
Quantitative VDRL	Mandatory	22.3	Not good
VDRL in liquor	Essential	2.5	Great
Quantitative VDRL in liquor	Mandatory	99	Bad
Changes in liquor	Mandatory	4.2	Great
Long bone x-ray changes	Mandatory	23.2	Not good
Child treatment	Mandatory	2.4	Great
Clinical diagnosis	Mandatory	2.3	Great

Source: Database from SINAN, provided by SES-Rio de Janeiro. NB: newborn; X-ray: radiography; VDRL: Venereal Disease Research Laboratory;

There was a progressive annual increase in absolute numbers and in incidence, from 6.3 in 2007 to 41.6 cases per 1,000 LB in 2018 (Table 3). The incidence according to sociodemographic variables showed that, in all years, the highest incidences were observed in adolescents, as opposed to women aged 35 years or older. In 2018, the incidence among adolescents was almost 10 times that of older women (Table 3).

**Table 3.** Annual incidence of syphilis (total and according to maternal sociodemographic variables) and prenatal testing in São Gonçalo, RJ, from 2007 to 2018.

	2007	2008	2009	2010	2011	2012	2013	2014	2015	2016	2017	2018
Total	6.3	4.4	4	4.1	4.9	9.5	15.4	18.5	27.4	32.3	41.2	41.6
Age range (years)												
10–19	9	8.6	8.4	8.2	7.8	19.9	28.8	31.2	49.2	62.9	68.3	90.6
20–34	5.7	3.5	2.9	3.2	4.5	7.2	13.3	16.8	24.8	29.2	39.9	37.5
≥35	5.5	3.3	1.8	2.5	2.4	4	4.6	5.9	10	8.1	13.8	9.7
Education (years of study)*												
<8	NA	NA	NA	NA	NA	NA	NA	27	62.5	81.4	86.9	122.8
≥8	NA	NA	NA	NA	NA	NA	NA	10	14.6	14.3	22.9	23.9
Skin color												
White	3	2.3	1.4	1.2	2.1	2.8	5.5	3.9	19.8	8.9	15.3	10.4
Black	43.5	42.8	30	48.8	23.1	22.5	25.6	29.9	62.8	91.1	131.9	87.6
Brown skin	4.8	3.2	4.8	5	5.8	12.6	20.7	25.3	27.6	41.5	46.3	51.1
Prenatal care (completion of any number of consultations)												
Yes	3.7	2.8	2.8	2.8	4	6.6	10.1	15.1	22.9	29.3	36.2	37.9
No	71	77.9	72.3	49.7	24.4	88.2	160.6	205.9	505.9	414.3	351.9	677.4

Source: Database from SINAN, provided by SES-Rio de Janeiro and SINASC.^14^ NA: not applicable. *Incidences were not estimated before 2014, due to the high number of ignored information.

As for maternal schooling, it was only possible to estimate incidences from 2014 to 2018, as the data were ignored in more than 50% of CS cases from 2007 to 2013. Using the cutoff point of 8 years of schooling (complete primary education), the incidence was always five times higher in women with low schooling (Table 3).

In all years, black women had the highest incidences, followed by brown women, while white women always presented the lowest values. Compared to white women, in 2018, the incidences of CS in black and brown women were eight and five times higher, respectively (Table 3).

Women who did not perform prenatal care always had a higher incidence than those who did, and in 2018, it was 17 times higher (Table 3).

Of the 2,420 cases of CS, 95.4% were LB, followed by 62 (2.6%) stillbirths and 48 (2%) abortions. The predominant final diagnosis was recent CS, with only six cases of the late form. Of the total number of NB with CS, 84.7% were asymptomatic and 36 died from the disease. Comparing the data with those of the SIM, the number of deaths was the same, but in different periods. Infant mortality from CS was upward in both information systems. In the first triennium, it was 11.6 (SIM) and 17.4 (SINAN) per 100,000 LB. In the last 3-year period, it reached 65.4 per 100,000 LB, a value in agreement between SIM and SINASC.

**Figure 1.**
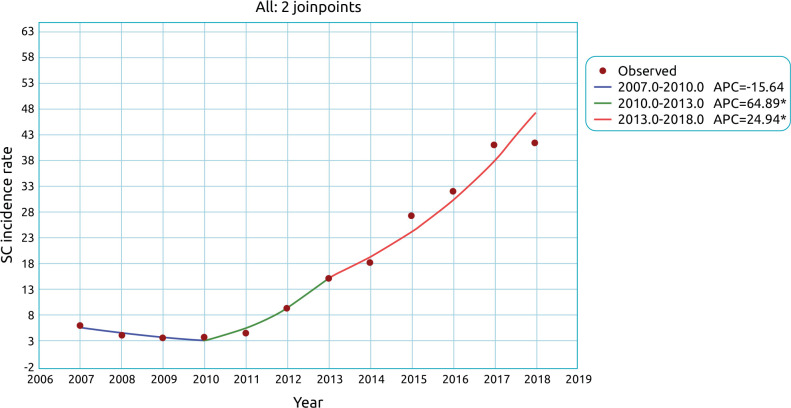
Time series of the incidence of congenital syphilis in São Gonçalo, RJ, from 2007 to 2018.

In the time series, the incidence of CS declined between 2007 and 2010, without significance; it increased annually by 64.9% from 2010 to 2013 and by 24.9% between 2013 and 2018, significantly (Table 4 and Figure 1).

**Table 4.** Annual percentage variation in the incidence of congenital syphilis, total and according to sociodemographic variables, and prenatal care, in São Gonçalo, RJ, from 2007 to 2018.

	2007	2018	Time course	Annual percentage change (95%CI)	Trend
Total	6.3	41.6	2007–2010	−15.6 (-32.4–5.3)	Stability
			2010–2013	64.9* (5.9–156.7)	Increase
			2013–2018	24.9* (13.2–37.9)	Increase
Age range (years)					
10–19	9	90.6	2007–2010	−1.1 (-32.6–45.1)	Stability
			2010–2018	37.3* (26.3–49.3)	Increase
20–34	5.7	37.5	2007–2009	−34.2 (-58.4–4.0)	Stability
			2009–2015	47.9* (33.5–63.9)	Increase
			2015–2018	17.5 (-6.5–47.7)	Stability
≥35	5.5	9.7	2007–2009	−41.5 (-72.4–24.0)	Stability
			2009–2018	24.6* (16.3–33.4)	Increase
Skin color					
White	3	10.4	2007–2018	23.6* (11.3–37.3)	Increase
Black	43.5	87.6	2007–2012	−14 (-31.9–8.8)	Stability
			2012–2018	33.5* (11.9–59.4)	Increase
Brown skin	4.8	51.1	2007–2018	31.6* (25–38.5)	Increase
Prenatal care					
Yes	3.7	37.9	2007–2010	−7.9 (-19.8–5.7)	Stability
			2010–2015	55.6* (42.6–69.8)	Increase
			2015–2018	18.6* (3.3–36.2)	Increase
No	71.1	677.4	2007–2011	−18.9 (-34.7–22.5)	Stability
			2011–2015	85* (20.9–183)	Increase
			2015–2018	5.6 (-310–61.6)	Stability
Education (years of study) **					
<8	27***	122.8	2014–2018	39.9* (9.8–78.3)	Increase
≥8	10***	23.9	2014–2018	24.5* (9.3–41.9)	Increase

Source: Database from SINAN, provided by SES-Rio de Janeiro and SINASC.^14^ 95%CI: 95% confidence interval. *p<0.05. **For schooling, only the period 2014–2018 was analyzed. *Indicates that the Annual Percent Chance (APC) is significantly different from zero at the alpha = 0.05 level. Final Selected Model: 2 Joinpoints.

*Indicates that the Annual Percent Chance (APC) is significantly different from zero at the alpha = 0.05 level. Final Selected Model: 2 Joinpoints.

Regarding the age group, the increase was statistically significant for all mothers, in different periods: 2010–2018 for adolescents; 2009–2015 for women aged 20–34 years; and, for older women, from 2009 to 2018. As for skin color, there was also statistical significance and the increase was more intense among black women (2012–2018), followed by brown and white women (the entire period). For prenatal care, there was stability at the beginning, followed by an increase, with varied intensity for those who performed it and very intense among those without prenatal care (2011–2015). Schooling was analyzed only from 2014 to 2018, for the quality of information, and the increase was more intense in those with less schooling (Table 4).

## DISCUSSION

The municipality of São Gonçalo, one of the few in the country with more than one million inhabitants, showed high and rising annual incidences of CS. The incidence of CS in 2018 was 41.6 per 1,000 NB, 80 times higher than the WHO target.^3^ Increases in the number of CS cases have been reported in Brazil, at national level^2,4,16^ or in local studies.^9,11,17,18,19,20,21,22,23,24^ In the country, the incidence of CS increased from 2.1 in 2009 to 8.2 per 1,000 LB in 2019,^2^ but was supplanted fivefold by that of São Gonçalo.

In 2019, the state of Rio de Janeiro had CS incidence of 20.1 per 1,000 LB and its capital, the city of Rio de Janeiro, of 13.8.2 In the state’s metropolitan region II, Niterói had an incidence of 23.6 per 1,000 LB in 2016,^11^ but São Gonçalo had an even greater magnitude.

The profile of sociodemographic inequalities related to CS was corroborated. The incidences were much higher in children of adolescents, women with low education, and those of black and brown skin color, confirming the situation of vulnerability of these populations.9,11,16,19,20,23,24

The incidence among women without prenatal care was much higher (677.4 per 1,000 NVA), but it was still high among those who had prenatal care, suggesting failures in the knowledge and practices of health professionals who care for these pregnant women, as shown in studies in the Southeast and Northeast regions.^7,8,16,25^ The main knowledge-related failures identified in those studies were related to serological tests (VDRL and treponemal tests), transmission rate by syphilis phase in pregnant women, CS criteria, and treatment of secondary and tertiary forms.^7,8,25^ As to practices, the most frequent failures were failure to request two serologies or request them after the deadline,^16,25^ insufficient frequency of cure control^25^, and inadequate treatment of the partner.^25^

In the period studied, the coverage of seven prenatal consultations or more barely reached 70% of pregnant women in São Gonçalo, and in recent years, the percentage has decreased, showing that, regarding the quantitative aspect, prenatal care was inadequate. Thus, the direct association of the number of consultations with the incidence of CS reported in the literature is strengthened.^6,9^ In the city of Rio de Janeiro, from 2011 to 2014, the variable that showed the highest association with CS was the low percentage of pregnant women with seven or more prenatal consultations.^9^ The access and continuity in the follow-up of pregnant women in Brazil are also linked to sociodemographic factors,^26,27^ perpetuating the inequality in maternal and child health outcomes. In the study by Benzaken *et al*,^27^ with national data, gestational syphilis more intensely affected adolescents, women of low education, and those mixed race and black. Despite the increased coverage of prenatal care, inequalities are detected in access and specifically in the performance of HIV and syphilis tests.^27,28^

Despite the majority of NBs being asymptomatic, CS mortality was high and rising in São Gonçalo. Noteworthy are infant mortality, which in the last triennium was much higher than that reported for Brazil in 2019, 5.9 per 100,000 NB, and that of the Southeast region, 6.9 per 100,000 NB.^2^ The number of infant deaths is consistent with that recorded in the SIM for the municipality, but stillbirths are in greater number in SINAN, showing the needs to qualify all health information systems and to expand the possibility of relationship between the databases. Furthermore, some variables of the NB had low completeness, data coinciding with that of a study in Minas Gerais,^21^ such as quantitative VDRL of liquor and alterations in radiographs of long bones, information of extreme importance for the follow-up of the NB with CS.

The temporal analysis of CS in São Gonçalo shows an annual increase of 64.9% between 2010 and 2013 and 24.9% between 2013 and 2018, confirming the upward trend of other regions of Brazil,^11,16,17,20–24^ but with even greater intensity. Of these studies, those that estimated the APV in similar time periods described smaller increases: in Niterói, from 2007 to 2016, the APV was 16%;^11^ in Pará, from 2007 to 2017, 12.3%;^17^ in Goiás, from 2007 to 2017, 16.8%;^18^ and in the Northeast, from 2008 to 2015, it was 19.9%.^20^ Only three explored the trend according to sociodemographic variables.^11,20,21^ Adolescents and women of brown or black color had a more accentuated increase in CS. Schooling had divergent results among the studies: in the Northeast^20^ and Santa Catarina,^23^ the increase in CS was lower or did not occur in women with lower schooling. As this study and that of Heringer *et al*.^11^ were carried out in the same metropolitan region of Rio de Janeiro, this may be a regional phenomenon. In the study from Santa Catarina, the authors argue that access to diagnosis may be greater among more educated women.^23^ There could also be methodological issues, such as missing information and the way it was worked out, but this aspect was not discussed in the articles.^19,23^

Two studies evaluated the trend according to prenatal care.^4,11^ Although the incidence was higher in women without prenatal care, the increasing trend was also identified in women who had prenatal care, denoting failures and probably a worsening in the quality of prenatal care.^4,11^ It was not possible to evaluate the impact of the shortage of benzathine penicillin on the increased incidence of CS, as already demonstrated in the city of Rio de Janeiro, but it is very likely that it happened.^10^

Specific actions in prenatal care should include monitoring of pregnant women, with a minimum of seven consultations; timely performance of tests according to the protocols of the Ministry of Health; strengthening the application of rapid tests for early diagnosis; active search for partners; and training of professionals in continuing education, especially those involved in prenatal care and during labor and birth.^27,28^

It is important to remember that CS and gestational syphilis are directly related to acquired syphilis, which is also on the rise in the country as a result of several factors such as increased risk behaviors, low condom adherence, association with social vulnerability, and stigma associated with any sexually transmitted infection.^1,2,29^ In São Paulo, an increase in acquired syphilis was observed mainly in the young and female population, pointing to the need for prevention and control of the disease even before pregnancy.^30^ Adding the increase in acquired syphilis to the persistence of failures in diagnosis and treatment during prenatal care, it explains, at least in part, the rise of CS.

The limitations of the study are related to the amount of ignored/empty information in SINAN, already reported in the literature,^11,23^ in addition to the prenatal variable, which in the research form is restricted to yes/no options, preventing the quantitative assessment of the number of consultations. Additionally, the absolute number of cases was small in some categories, generating wide confidence intervals and reducing the accuracy of some estimates, but without annulling the significance of most of the results regarding the growth of the annual incidence of CS.

Even considering these limitations, the results highlighted the characteristics and severity of CS in this municipality in the state of Rio de Janeiro, whose population is only exceeded by that of the capital. The importance of local studies is reinforced, due to the variation in the pattern of distribution of CS, which may reflect different models of organization of health care, as well as the possibility of identifying inequalities and inequities in different populations, contributing to the reduction of the occurrence and severity of syphilis.

The syphilis epidemic was corroborated in another municipality in Brazil,^29^ with a growing trend and the same social inequality pointed out in other places of the country. Although out of the usual reach of health professionals, it is necessary to ratify that broader measures that reduce social inequalities will impact on the occurrence and behavior of CS.

## References

[1] Korenromp EL, Rowley J, Alonso M, Mello MB, Wijesooriya NS, Mahiané SG (2019). Global burden of maternal and congenital syphilis and associated adverse birth outcomes-Estimates for 2016 and progress since 2012. PLoS One..

[2] Brazil - Ministério da Saúde [homepage on the Internet] (2020). Departamento de Doenças Crônicas e Infecções Sexualmente Transmissíveis. Boletim Epidemiológico – Sífilis 2020.

[3] World Health Organization. Pan American Health Organization [homepage on the Internet] (2019). New generations free of HIV, syphilis, hepatitis B, and chagas disease: EMTCT plus in the Americas, 2018.

[4] Oliveira VS, Rodrigues RL, Chaves VB, Santos TS, Assis FM, Ternes YM (2020). Aglomerados de alto risco e tendência temporal da sífilis congênita no Brasil. Rev Panam Salud Publica..

[5] Estado do Rio de Janeiro [homepage on the Internet] Secretaria de Estado de Saúde do RJ (SES/RJ). Subsecretaria de Vigilância em Saúde. Gerência de DST, HIV/AIDS, Sangue e Hemoderivados. Informe epidemiológico. Sífilis Adquirida, Materna e Congênita.

[6] Domingues RM, Saracen V, Hartz ZM, Leal MD (2013). Congenital syphilis: a sentinel event in antenatal care quality. Rev Saude Publica..

[7] Domingues RM, Hartz ZM, Leal MC (2012). An evaluation of action taken to control syphilis and HIV in public-sector prenatal care in the municipality of Rio de Janeiro, Brazil. Rev Bras Saúde Mater Infant..

[8] Domingues RM, Laurie L, Sarraceno V, Leal M (2013). Treatment of syphilis during pregnancy: knowledge, practices and attitudes of health care professionals involved in antenatal care of the Unified Health System (SUS) in Rio de Janeiro City. Cienc Saude Coletiva..

[9] Reis GJ, Barcellos C, Pedroso MM, Xavier DR (2018). Intraurban differentials in congenital syphilis: a predictive analysis by neighborhood in the city of Rio de Janeiro, Brazil. Cad Saude Publica..

[10] Braga JU, Araujo RS, Souza AS (2021). The shortage of Benzathine Penicillin and its impact on congenital syphilis incidence: an ecologic study in the City of Rio de Janeiro. Clin Infect Dis..

[11] Heringer AL, Kawa H, Fonseca SC, Brignol SM, Zarpellon LA, Reis AC (2020). Inequalities in congenital syphilis trends in the city of Niterói, Brazil, 2007-2016. Rev Panam Salud Publica..

[12] Malta DC, Almeida MF, Ribeiro AL (2021). Estimates in small geographic areas: a necessary step towards reducing health inequalities. Rev Bras Epidemiol..

[13] Brazil - Ministério da Economia [homepage on the Internet] Instituto Brasileiro de Geografia e Estatística (IBGE). Cidades. Rio de Janeiro.

[14] Estado do Rio de Janeiro [homepage on the Internet]. Secretaria de Estado de saúde do Rio de Janeiro (SES/RJ) Informações de Saúde. Sistema de Informações sobre Nascidos Vivos.

[15] Romero DE, Cunha CB (2007). Evaluation of quality of epidemiological and demographic variables in the Live Births Information System, 2002. Cad Saude Publica..

[16] Domingues RM, Leal Mdo C (2016). Incidence of congenital syphilis and factors associated with vertical transmission: data from the Birth in Brazil study. Cad Saude Publica..

[17] Carmo BA, Santos DF, Hayase KA, Santos MM, Naiff GR, Botelho EP (2020). Congenital syphilis in the Brazilian Amazon region: temporal and spatial analysis. Rev Eletr Enferm..

[18] Nunes PS, Guimarães RA, Rosado LE, Marinho TA, Aquino ÉC, Turchi MD (2021). Temporal trend and spatial distribution of syphilis in pregnancy and congenital syphilis in Goiás, Brazil, 2007-2017: an ecological study. Epidemiol Serv Saude..

[19] Oliveira LR, Santos ES, Souto FJ (2020). Syphilis in pregnant women and congenital syphilis: spatial pattern and relationship with social determinants of health in Mato Grosso. Rev Soc Bras Med Trop..

[20] Souza CD, Machado MF, Correia DS, Carmo RF, Cuevas LE, Santos VS (2020). Spatiotemporal clustering, social vulnerability, and risk of congenital syphilis in northeast Brazil: an ecological study. Trans R Soc Trop Med Hyg..

[21] Alves PI, Scatena LM, Haas VJ, Castro SS (2020). Temporal evolution and characterization of congenital syphilis cases in Minas Gerais, Brazil, 2007-2015. Cienc Saude Coletiva..

[22] Vescovi JS, Schuelter-Trevisol F (2020). Increase of incidence of congenital syphilis in Santa Catarina state between 2007-2017: temporal trend analysis. Rev Paul Pediatr..

[23] Rocha RP, Magajewski FR (2018). Historic and epidemiologic tendency of congenital syphilis in the state of Santa Catarina between 2007-2016. Arq Catarin Med..

[24] Teixeira LO, Belarmino V, Gonçalves CV, Mendoza-Sassi RA (2018). Temporal trend and spatial distribution of congenital syphilis in the state of Rio Grande do Sul between 2001 and 2012. Cienc Saude Coletiva..

[25] Rodrigues DC, Domingues RM (2018). Management of syphilis in pregnancy: knowledge and practices of health care providers and barriers to the control of disease in Teresina, Brazil. Int J Health Plann Manage..

[26] Mallmann MB, Boing AF, Tomasi YT, Anjos JC, Boing AC (2018). Evolution of socioeconomic inequalities in conducting prenatal consultations among Brazilian parturient women: analysis of the period 2000-2015. Epidemiol Serv Saude..

[27] Benzaken AS, Pereira GF, Cunha AR, Souza FM, Saraceni V (2019). Adequacy of prenatal care, diagnosis, and treatment of syphilis in pregnancy: a study with open data from Brazilian state capitals. Cad Saude Publica..

[28] Freitas CH, Forte FD, Galvão MH, Coelho AA, Roncalli AG, Dias SM (2019). Inequalities in access to HIV and syphilis tests in prenatal care in Brazil. Cad Saude Publica..

[29] Santos M, Lopes AK, Roncalli AG, Lima KC (2020). Trends of syphilis in Brazil: a growth portrait of the treponemic epidemic. PLoS One..

[30] Luppi CG, Tayra A, Domingues CS, Gomes SE, Pinto VM, Silva MA (2020). Syphilis in the state of São Paulo, Brazil, 2011–2017. Rev Bras Epidemiol..

